# Long-Term Feeding of Dairy Goats with 40% Artichoke by-Product Silage Preserves Milk Yield, Nutritional Composition and Animal Health Status

**DOI:** 10.3390/ani13223585

**Published:** 2023-11-20

**Authors:** Paula Monllor, Jihed Zemzmi, Raquel Muelas, Amparo Roca, Esther Sendra, Gema Romero, José Ramón Díaz

**Affiliations:** 1 Department of Agro-Food Technology, Escuela Politécnica Superior de Orihuela (EPSO), Instituto de Investigación e Innovación Agroalimentaria y Agroambiental (CIAGRO-UMH), Universidad Miguel Hernández de Elche (UMH), Ctra. de Beniel, Km 3.2, 03312 Orihuela, Spain; pmonllor@umh.es (P.M.); raquel.muelas@umh.es (R.M.); aroca@umh.es (A.R.); esther.sendra@umh.es (E.S.); 2 Servicio de Nutrición y Bienestar Animal, Department of Ciencia Animal y de los Alimentos, Universidad Autónoma de Barcelona, 08193 Bellaterra, Spain; jihed.zemzmi@uab.cat

**Keywords:** urea, mineral profile, fatty acid profile, circular economy

## Abstract

**Simple Summary:**

The rational use of agriculture and agro-industrial by-products in ruminant nutrition contributes to greater respect for the environment in addition to a better final product quality. Artichoke crops are widespread in the Mediterranean region but mostly not properly valued. Previous studies carried out with artichoke by-product silage at 25, 40 and 60% inclusion in dairy goat feed for one month showed that 40% inclusion would be a good option without any harm to milk yield and composition or animal health status. Therefore, it is worth studying the effect of feeding animals with 40% artichoke by-product silage during a full lactation period. No negative effects were observed on animal performances and productivity, allowing us to reduce agroindustry wastes and offering a long-term preserved by-product that promotes a sustainable circular economy and the production of animal products with high nutritional value, like goat milk.

**Abstract:**

The aim of this work is to study the effect of 40% inclusion of artichoke by-product silage (AB) in dairy goat diets on milk yield, composition and animal health status during a full lactation period compared to an isoenergetic and isoproteic mixed ration based on alfalfa hay and a cereal and legume mixture. Milk yield was not affected by the dietary treatments, and neither was body weight. AB treatment reduced whey protein (0.38 vs. 0.42%, *p* < 0.05) and milk urea concentrations (687 vs. 773 mg/L, respectively, *p* < 0.001), and did not affect total true protein (3.22 vs. 3.24% *p* > 0.05) or other macro-composition variables. AB treatment showed higher milk concentrations of Ca (*p* < 0.05), Mn (*p* < 0.01), Cu (*p* < 0.01) and Zn (*p* < 0.001) compared to the control group (C). Slight differences were observed in milk fatty acid profile without any negative effects (*p* > 0.05) on the blood cholesterol and glucose of goats. The AB group reduced blood urea due to its high dietary total phenol content. However, it had a positive effect on β-hydroxybutyrate (*p* < 0.05) and nonesterified fatty acids (*p* > 0.05). It was concluded that 40% artichoke by-product inclusion in dairy goat feed for the whole lactation period (23 weeks) is a sustainable solution, reducing feeding cost by 12.5% per kg of dry matter, contributing to a better circular economy without any negative repercussions on the productivity and health of Murciano–Granadina dairy goats.

## 1. Introduction

The transition toward a circular economy is one of the main priorities of the 8th Environment Action Program to 2030, making waste management, recycling and re-use crucial challenges in the present day [1]. Animal feeding represents more than 60% of the livestock production costs, consuming natural resources such as water, land and fossil fuel. Moreover, these products usually require long-distance transportation to farms, which may impair their nutritional quality but also increase costs and environmental contamination. Thus, the use and valorisation of locally produced by-products in animal production feeding is a sustainable environmentally friendly idea that may reduce costs and greenhouse gas emissions [2,3]. Hence, the use of industrial by-products will turn wastes into raw materials with high nutritional quality for livestock feeding and therefore into meat and milk for human consumption [4]

Spain, along with Italy, is one of the main artichoke-producing countries in the European Union. National production is concentrated in the south of the province of Alicante and the Region of Murcia, where in 2018 more than 9000 ha of artichoke were cultivated, with a total production of more than 120,000 t. The remnants of the artichoke plants left in the field after harvesting is equivalent to 11 t of forage per hectare [5]. On the other hand, packing and freezing industries only use the central 50 per cent of the artichokes; the rest is made up of bracts, stems and inedible parts. All this entails a great availability of these by-products that can be used for animal feeding.

As reported by García-Rodríguez et al. [6], the majority of these by-products are characterized by a moderate neutral detergent fibre content (400–600 g/kg), medium metabolizable energy (1.72–1.90 Mcal/kg DM) and a medium-to-high crude protein concentration (160–200 g/kg), and therefore they are potential candidates for replacing forage and alfalfa and soybean-based concentrate in the diet. Moreover, artichoke by-product silage is able to be preserved for 200 days according to [7]; it has a suitable fermentative property that guarantees the fulfilment of nutritional needs and the safety required for inclusion in small ruminant feeding [7,8]. 

In a previous work [9], different levels (25, 40, 60%) of inclusion of artichoke by-product silage (AB) made from in-field plant leftovers after harvest were tested during one month in the feeding of Murciano–Granadina goats, and it was concluded that AB could be introduced up to a maximum level of 40%. Additionally, it was observed that it ameliorated the milk lipid profile due to its higher content of polyunsaturated fatty acids (4.37%) and lower atherogenicity (1.90) and thrombogenicity indices (3.05) [9]. Artichoke plant was tested for a short-term period of five weeks [9] and later for a long-term experiment of 23 weeks [10]; the results showed that its inclusion in dairy goat feeding to a maximum of 40% is a great solution to reduce feeding costs without harming animal health and productivity. 

The present study aims to focus on artichoke industry by-products (mainly bracts and stems), whose composition significantly differs from that of the previously tested material (artichoke plant). The ensiled by-product was included at a level of 40% in the diet of Murciano–Granadina dairy goats and the effect of the diet on health status and milk quality was assessed during a complete lactation.

## 2. Materials and Methods

### 2.1. Animals and Facilities

Murciano–Granadina goats from the experimental farm of the Miguel Hernández University were allocated with a straw bed; they had access to outdoor yards, free access to water and enough feeder space for all animals (at least 35 cm/animal). Animals were fed twice a day, at 8:00 and 14:00, and milked once a day (Casse milking parlour, 2 × 12 × 12, GEA, Bönen, Germany), as is usual in the region. The experiment was carried out between the months of May and October. This study was approved and authorized by the Ethical Committee of Experimentation of the Miguel Hernández University (code UMH.DTA.GRM.01.21), authorized by the competent public body (Conselleria de Agricultura, Desarrollo Rural, Emergencia climática y transición ecológica of Generalitat Valenciana, Spain, code 2021/VSC/PEA/0169).

### 2.2. Experimental Design

From a group of 80 goats at the onset of lactation (4th week) fed with a conventional diet (control, C), a pre-experimental individual sampling was performed, and 54 animals were selected, with an average body weight (BW) of 43.5 ± 8.70 kg, an average milk yield of 2.34 ± 0.81 kg/day and a somatic cell count (SCC) of 2.16 ± 0.52 Log cells/mL. Animals were divided into two groups with homogeneous characteristics regarding the commented variables. Each group was assigned to one treatment: control (C), which represents the conventional diet of the region (alfalfa hay and a mixture of grains), and one treatment ration that included 40% (on a dry-matter basis) of artichoke by-product silage (AB) in a total mixed ration (TMR). AB was prepared following the patent registered by Monllor et al. [8].

Silage was manufactured with a baler–wrapper (Agronic MR 820, Haapavesi, Finland), with a weight of 300 kg and without additives, according to the work published by Monllor et al. [10]. All feeding rations were calculated according to formulation recommendations by Fernández et al. [11]. Diets were isoenergetic and isoproteic and the daily amount offered was 2.22 kg DM/day. AB silage had a lower cost than other feedstuffs used in animal feeding (42 EUR/t DM). Table 1 and Table 2 show the diet composition and costs of both diets. Feeding was performed in a group to avoid the stress caused by the individual confinement of the animals during a long-term study (6 months), as was the case with Nudda et al. [12], due to the gregarious behaviour of goats [13]. This experimental design allows the average food consumption of the animals to be determined, although not the exact amount of individual feed intake nor ingested nutrients. However, the facilities and devices present on the farm allowed the collection of individual milk samples.

Once the pre-experimental sampling was complete (week 4 of lactation), the experiment lasted 23 weeks. The first three weeks for each group served as time to adapt to their diet. In the following 20 weeks, data on BW and milk yield were taken, and blood and individual milk samples were collected for a subsequent laboratory analysis every 5 weeks; sample 1 at week 7, sample 2 at week 12, sample 3 at week 17, sample 4 at week 22 and finally sample 5 at week 27, and therefore obtaining five samples in total during the whole experiment. In addition, the same week of individual sampling, after the milking of each experimental group, milk samples from the pool of every experimental group were collected on three consecutive days for an analysis of the mineral and lipid profile. 

### 2.3. Variables Analysed

BW (kg) was determined by weighting animals with a precision scale of 100 g (APC, Baxtran, Zafra, Spain). Feed consumption data were taken from two consecutive days in each sampling week and calculated as the average of the difference between the feed amount offered and refused, determining the dry matter by dehydration in an oven at 105 °C for 48 h of a representative sample of the feed amount refused by the animals for each treatment. Representative samples were taken from silage and ration at the start of the experiment for a subsequent laboratory analysis. The composition of the rations was determined in a similar way to Monllor et al. and Monllor et al. [14,15] using Association of Official Agricultural Chemists (AOAC) methods [16] for dry matter (DM, g/kg; method 930.5), organic matter (OM, g/kg DM; method 942.05), ether extract (EE, g/kg DM; method 920.39) and crude protein (CP, g/kg DM; method 984.13). The contents of neutral detergent fibre (NDF, g/kg DM), acid detergent fibre (ADF, g/kg DM) and acid detergent lignin (ADL, g/kg DM) were analysed according to Van Soest et al. [17]. The total polyphenol content (TP, g/kg DM) was determined via the Folin–Ciocalteu method reported in Kim et al. [18]. The proportion of short-chain volatile fatty acids (VFAs, g/kg DM)—acetic, propionic and butyric acid, also including lactic acid and ethanol—were determined with high-performance liquid chromatography (HPLC) (Agilent 1200 and Supelcogel C-610H column: 30 cm × 7.8 mm ID; [19]). The apparent in vitro dry matter digestibility (*IV*DMD, g/kg DM) was determined in duplicate with the method of Menke and Steingass [20]. An analysis of the fatty acid profile in the diets was carried out via direct methylation on the lyophilized samples, without prior extraction of the fat, according to Kramer et al. [21]. Methylated fatty acid esters (FAMEs) from diets were identified and quantified with a gas chromatograph (GC-17A Shimadzu, Kioto, Japan) coupled with a flame ionization detector (FID) equipped with a capillary column (CP Sil 88 100 m × 0.25 mm internal diameter and 0.20 µm internal coverage, Agilent, Santa Clara, CA, USA). A FAME standard mix (18912-1AMP, Sigma-Aldrich, Saint Louis, MO USA) was used to identify the fatty acids present in the samples.

For the analysis of dietary and milk minerals, the same procedures were followed as in Monllor et al. [15], with a previous digestion of the samples performed according to González Arrojo et al. [22]. Na, Mg, K, Ca, P, S (g/kg DM) and Se, Zn, Cu, Fe and Mn (mg/kg DM) were determined via prior microwave digestion (Ethos Easy, MilestoneSlr, Sorisole, Italy) and identified with an ICP-MS octupole chromatograph (Agilent 7500 Reaction System, Santa Clara, California, USA), using an internal standard.

The dairy yield and macrocomposition were determined similarly to Monllor et al. [14]. The milk production of each animal (kg/day) was recorded during milking using a Lactocorder^®^ device (WMB AG, Balgach, Switzerland), which collected a 100 mL individual sample for subsequent analyses. The milk macrocomposition (fat, protein, useful dry extract, UDM; true protein, casein, whey protein, lactose, total solids, TSs; nonfat total solids, NFTSs; ash; %) and urea content (mg/L) was analysed with medium infrared spectroscopy (MilkoScan™ FT2, Foss, Hillerød, Denmark) calibrated for goat milk. The somatic cell count (SCC, Log cell/mL) was determined via an electronic fluoro-optical method (DCC, DeLaval, Sweden). The fat-corrected milk yield was calculated according to the Gravert equation [23]: fat-corrected milk (FCM) (3.5%) = 0.433 × yield (kg/day) + 16,218 × fat yield (kg/day), and fat- and protein-corrected milk yield according to the equation of Schau and Fet [24]: FPCM = yield (kg/day) × (0.337 + 0.116 × fat (%) + 0.060 × protein (%)).

Analysis of the milk fatty acid profile was carried out in duplicate, with an extraction using the Folch method with some variations reported in Romeu-Nadal et al. [25] and a subsequent methylation according to the method of Nudda et al. [26], similar to Monllor et al. [14]. The chromatograph, the column and the FAME mix for the identification of the milk fatty acids were the same as those used in the diets. The indices related to the nutritional quality of milk fat were calculated—the Atherogenicity Index (AI) and Thrombogenicity Index (TI)—according to Ulbricht and Southgate [27], and the Desaturase Index (DI) for C14:0, C16:0 and C18:0, according to Lock and Garnsworthy [28]. 

On the same day that the milk sampling was performed, blood samples were taken from fasting animals for an analysis of glucose, urea, β-hydroxybutyrate (BHB), cholesterol, nonesterified fatty acids (NEFAs) and haematocrit, similarly to Monllor et al. [9]. Blood samples were analysed by enzymatic spectrophotometry. For glucose and cholesterol (mg/dL), a glucose oxidase/peroxidase kit was used (Refs. 11,503 and 11,505, Biosystems, Barcelona, Spain); for urea (mg/dL), the kinetic method GN 10,125 developed by Gernon was used (Spain); for the BHB (mmol/L), the Ranbut D-3-Hydroxybutyrate kit (RB 1007, Randox, Crumlin, UK) was used, and for the NEFAs (mmol/L), an enzymatic–spectrophotometric method FA 115 (Randox, Crumlin, UK) was used. The haematocrit (%) was determined with a microhaematocrit.

### 2.4. Statistical Analysis

SCC values were transformed into logarithm base 10 to carry out the statistical analysis. A normality test was performed on data (PROC UNIVARIATE. SAS v9.2, 2012) and the result was that all variables complied with normality and variance homogeneity. The variables obtained from individual animals were analysed according to a mixed linear model with repeated measures (PROC GLIMMIX. SAS v9.2, 2012), introducing into the model the covariate of the data obtained in the pre-experimental sampling according to the following equation:

Y = µ + Di + Wj + Di × Wj + covY0 + Ak + e,(1)

where Y is the dependent variable, µ is the intercept, Di is the fixed effect of the diet (i = C, AB), Wj is the fixed effect of the lactating week (j = 7, 12, 17, 22, 27), Di × Wj is the interaction of the diet with the lactating week, covY0 is the effect of the value of Y in control 0, Ak is the random effect of the animal and e is the residual error. For each variable, the covariance model that presented lower AIC and BIC statistics was used.

In the case of the feed consumption, milk lipid and mineral profile variables, an ANOVA (PROC. GLM, SAS v9.2, 2012) was performed, similar to the previous one, except that the random effect of the animal was not considered.

## 3. Results

No significant differences (*p* > 0.05) between groups were observed in the pre-experimental sampling (fourth week of lactation) for any of the variables analysed (body weight, milk yield and composition, SCC and milk mineral and fatty acid profile). 

### 3.1. Body Weight and Milk Performance

**Figure 1:**
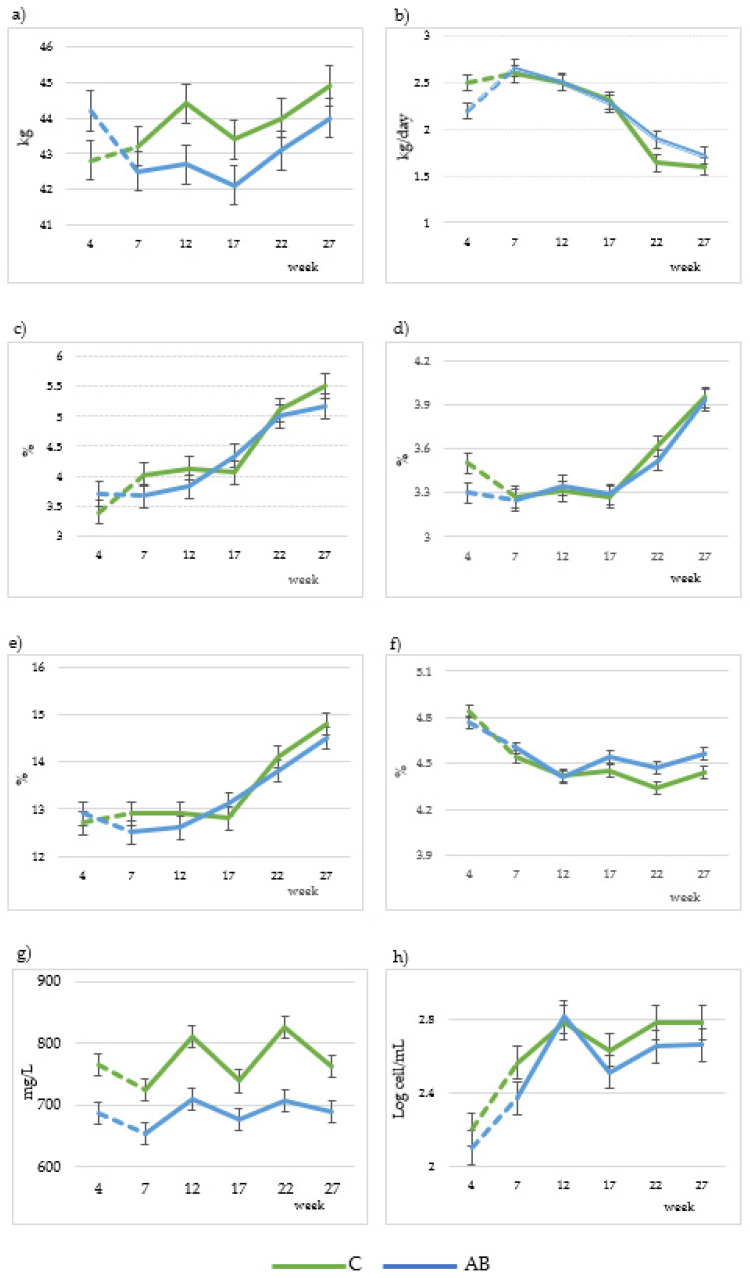
Comparison of changes in body weight (**a**), milk yield (**b**), milk fat (**c**), milk protein (**d**), total solids (**e**), ash (**f**), milk urea (**g**) and somatic cell count (**h**) in goat milk (22 goats per group) from 7th to 27th lactation weeks. C: Control diet; AB: diet that includes artichoke by-product silage. Dashed lines indicate the adaptation time to the diets.

The diet effect was not significant for most of the studied variables, having a significant effect only for whey protein, ash and milk urea. Sampling time (number of weeks in the diet) was significant for all the studied variables, with the interaction with treatment only being significant for whey protein and lactose. For both treatments, BW increased significantly (*p* = 0.033) from the 7th to the 12th week of the experiment (43.2 and 42.5 to 44.4 and 42.7 kg for C and AB, respectively), decreased (*p* = 0.021) during the 17th week (43.4 and 42.1 kg for C and AB diets, respectively) and then increased during the 22nd and the 27th weeks to reach a final BW of 44.9 and 44.0 kg for C and AB, respectively (*p* = 0.005) (Figure 1a). The C group showed a higher non-significant average BW (*p* > 0.05 Table 3). The average feed consumption was higher in the C group (2.00 ± 0.11 and 1.7 ± 0.13 kg DM/day, respectively, for C and AB groups). Both the C and AB groups reduced mid-lactation feed consumption: 160 g DM/day less between the 12th and 17th weeks in the C group and 180 g DM/day less between the 17th and the 22nd weeks in the AB group.

**Figure 2:**
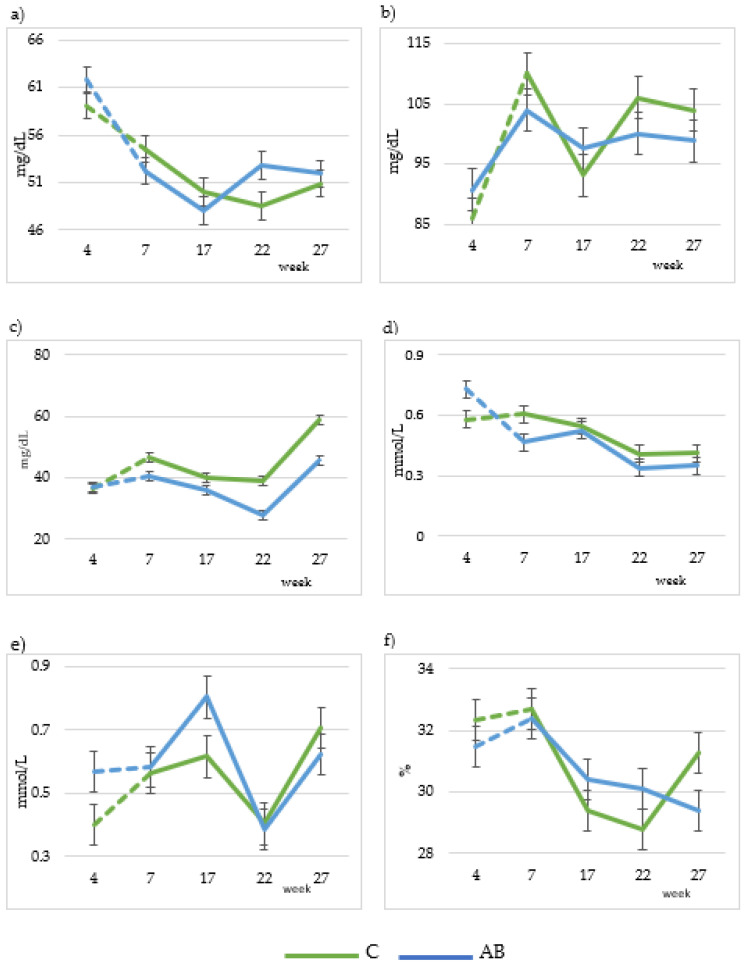
Changes in plasmatic metabolites (24 goats per group) throughout the experiment: glucose (**a**), cholesterol (**b**), urea (**c**), BHB (**d**), NEFA (**e**) and haematocrit (**f**). C: Control diet; AB: diet that includes artichoke by-product silage. Dashed lines indicate the adaptation time to the diets.

Milk yield was not affected by the dietary treatment (*p* > 0.05, Table 3), showing a regular decrease (*p* < 0.001) in both groups throughout the experiment (Figure 2b). With respect to FCM and FPCM, both treatments showed similar mean values (Table 3). The values of both treatments, C and AB, decreased gradually as lactation progressed (*p* < 0.001). With regard to milk macrocomposition, both treatments showed similar mean values for fat, protein, SCC, UDM, TS, NFTS, true protein, casein and lactose. Whey protein, ash and milk urea values were significantly (*p* < 0.05) lower in the AB group. Additionally, we observed a gradual increase in fat, protein, TS and SCC during the lactation periods (Figure 1c–e,h, respectively) for both experimental groups. Lactose decreased (Figure 1f) and milk urea fluctuated (*p* < 0.001) during the lactation period (Figure 1g) in both treatments.

### 3.2. Milk Mineral Profile

Milk mineral profile is presented in Table 4. The AB group showed the highest milk concentrations in Ca, Mn, Cu, Zn (*p* < 0.05). The C group showed slightly higher (*p* < 0.05) Mg and Se content than AB. The stage of lactation (sampling week) effect was significant (*p* < 0.001) for all the minerals studied, with the interaction only being significant for Ca and Zn (*p* < 0.05). Regarding the evolution of mineral contents during the lactation period, Na, Mg, P, S, Ca and Se contents increased in the 12th week, decreased in the 17th week and then increased gradually in the 22nd and the 27th weeks of lactation, presenting the highest (*p* < 0.05) values in the 22nd week for both experimental groups. Cu concentration decreased gradually (*p* < 0.001) during lactation. Zn content was almost stable until the 17th week of lactation and then increased gradually until the 22nd week, showing a significant higher (*p* < 0.001) value in the AB group, which made the interaction between the dietary treatment and the lactation week significant (*p* < 0.001). K and Fe content fluctuated between the 7th and the 27th weeks, reaching its maximum at the 12 week of lactation.

### 3.3. Milk Fatty Acid Profiles

Few fatty acids showed significant differences due to the treatment (Table 5), being slight and mainly biologically not relevant. The most important saturated fatty acid proportion with significant differences between the C and AB groups was palmitic acid (C16:0, 23.9, 24.5% in C and AB groups, respectively, *p* < 0.001), showing a small difference. The most abundant monounsaturated fatty acids (Table 5) were oleic acid (C18:1 c9, 18.9, 19.6% in C and AB groups, respectively) and vaccenic acid (C18:1 t11, 2.13, 1.37% in C and AB groups, respectively). The most abundant polyunsaturated fatty acids (Table 5) were found to be linoleic acid (C18:2n6, 2.58, 2.59% in C and AB groups, respectively, *p* > 0.05), and rumenic acid (CLA c9, t11, 0.843, 0.602% in C and AB groups, respectively, *p* < 0.001) 

Regarding fatty acid groups and indices related to cardiovascular health (Table 6), the dietary treatment significantly affected (*p* < 0.01), although slightly, the concentrations of SFA, MUFA, PUFA, UFA, OBCFA, ∑CLA, MCFA and LCFA, the ratios of SFA/UFA and n6/n3, and the AI, TI and DI C18:0 indices. The AB group presented higher concentrations of SFA, OBCFA and MCFA (68.6 and 67.4, 3.46 and 3.29, 41.6 and 40.8 for AB and C groups, respectively), in addition to higher ratios of SFA/UFA and n6/n3 (2.19 and 2.08, 17.7 and 15.6 for AB and C groups, respectively) and higher AI and TI indices (2.09 and 2.02, 3.20 and 3.045 for AB and C groups, respectively). Instead, the C group presented higher (*p* < 0.01) concentrations of MUFA, PUFA, UFA, ∑CLA, LCFA and n3 (27.5 and 26.6, 4.93 and 4.64, 32.5 and 31.2, 0.954 and 0.726, 45.3 and 44.3, and 0.193 and 0.164 for C and AB groups, respectively) and higher DI C18:0 (1.84 and 1.75 for C and AB groups, respectively).

### 3.4. Plasmatic Metabolite Profiles

Plasma metabolite profiles are shown in Table 7. Urea concentration decreased (*p* < 0.001) in the AB group (46.1 and 37.5 mg/dL for urea concentration in C and AB groups, respectively). Similarly, BHB concentration decreased (*p* < 0.05) in the AB group (0.49 and 0.42 mmol/L for C and AB groups, respectively). The lactation week significantly affected all variables. Glucose concentration decreased in both groups until the 17th week and then increased in the AB group while it decreased in the C group during the 22nd week, and finally decreased in the AB group and increased in the C group in the 27th week, reaching a slightly lower value compared to the initial value (Figure 2a). Cholesterol concentration had a similar evolution in both the C and AB groups; it oscillated, reaching a lower value as compared to the initial value (Figure 2b). Blood urea concentration decreased until the 22nd week and increased in the 27th week, reaching its highest value in both groups (Figure 2c). BHB decreased for both groups during the lactation period (Figure 2d). NEFA concentration oscillated in both groups and reached a slightly higher value at week 27 as compared to week 4 (Figure 2e). Finally, haematocrit concentration decreased gradually till the 22nd week of lactation in both groups, except for week 27, when it increased in the C group and decreased in the AB group (Figure 2f).

## 4. Discussion

### 4.1. Body Weight and Milk Performance

Similar to previous results reported by Monllor et al. [10], a reduction in the BW was observed in the first sampling week (7th week of lactation), which was due to the low intake capacity and the highest lactation needs at the same time. BW increased linearly during the 4th and the 5th samples due to the decrease in milk yield and the increase in ingestion capacity, which resulted in a recovery of feed consumption without differences due to the dietary treatment (*p* > 0.05). Values from average feed consumption were adequate for dairy goats and similar to those reported by Monllor et al. [9] when studying artichoke by-product silage for a short time (2.21 kg DM/day for control and 1.73 kg DM/day for ABS) and to those published by Criscioni and Fernández [29] (1.7 kg DM/day) in Murciano–Granadina goats.

AB animal milk yield and its main composition were not affected negatively by the 40% inclusion of artichoke by-product silage. Milk urea concentration values decreased in the AB group compared to the C group, being higher than those obtained by Monllor et al. [9] when studying artichoke by-product silage for 5 weeks of lactation, but comparable to those obtained by Monllor et al. [10] when the study was conducted over a whole lactation period including artichoke plant by-product. The main determinants of urea formation in milk are the amount of crude protein intake and the ratio between protein and energy proportion [30]. However, both diets were designed to be isoenergetic and isoproteic. Another factor that may affect milk urea concentration is NDF content (369 vs. 444 g/100 g DM for C and AB groups, respectively) [29]. According to Bonanno et al. [31], NDF and milk urea concentration are negatively correlated, which explains its lower value in then AB group. 

### 4.2. Milk Mineral Profile

Slight differences were observed in the diet and in milk with regard to the mineral content. The AB group showed higher Ca concentration in milk (+34 mg/kg, *p =* 0.05), which favours the coagulation and curd firmness of milk, although this treatment showed the lowest content in the diet (−2.63 g/kg DM). This can be explained by a mobilization of bone Ca to maintain its level in milk that also resulted in a higher Ca/P ratio. Equivalently, AB group milk showed higher Mn and Cu, despite their lower concentration in the diet compared to the C diet. The higher concentration of Zn in AB group milk is probably due to its higher concentration in the diet. However, Se and Mg concentrations were higher in the C diet and the C group milk. It seems that artichoke by-product silage enhances the milk mineral profile of dairy goats without any negative impact on Ca and P concentrations, which is very important in cheese formation [32]. However, Ca supplementation must be considered to avoid Ca deficiencies in the subsequent lactations.

### 4.3. Milk Fatty Acid Profile

OBCFA proportions were around 3.3 and 3.5% (Table 6), similar to previous values published by Monllor et al. [10] when studying the effect of artichoke plant and broccoli by-product silage for a full lactation on Murciano–Granadina goats in a similar experimental condition. However, they seem to be higher than those reported by Monllor et al. [9] when studying different doses of artichoke by-product silage on Murciano–Granadina goats for a 5-week period. Moreover, OBCFAs in milk, as well as their isomers of tridecanoic acid (iso C13:0), tetradecanoic acid (iso C14:0), pentadecanoic acid (C15:0, iso C15:0 and anteiso C15:0), hexadecanoic acid (iso C16:0) and heptadecanoic acid (C17:0, iso C17:0 and anteiso C17:0), are synthetized de novo by ruminal bacteria [33]. Their proportion is correlated positively with NDF level in the diet due to a higher production of volatile fatty acid. In the case of the present study, the AB group diet had a higher content of NDF and therefore a higher OBCFA, iso C13:0, iso C14:0, iso C15:0, anteiso C15:0, C15:0, C15:0, iso C16:0 and iso C17:0. The lower concentration of rumenic, vaccenic and linoleic acid in AB animals’ milk may be explained by the lower amount of linoleic acid (−3.7 g/100 g of total fatty acids) in the diet, which is a precursor of the biohydrogenation (BH) process responsible for these fatty acids’ formation [34]. Otherwise, linolenic acid, which is also a precursor of BH, was slightly higher (+0.77 g/100 g of total fatty acids) in the AB diet but with no effect on the aforementioned acids, probably because there was a much higher amount of linoleic acid in the C diet as in Monllor et al. [10]. Short-chain fatty acids are reported to be a crucial player in the organoleptic properties of milk [35], as published by Monllor et al. [9]. Consequently, artichoke by-product silage is not expected to modify milk flavour. 

### 4.4. Plasmatic Metabolites Profile 

Values from plasmatic metabolites were similar to those obtained by Monllor et al. [3] and Monllor et al. [10]. Low blood urea is correlated with high TP content in the diet [36]. High TP in the AB diet would reduce protein rumen digestibility, forming non-degradable complexes in the rumen, reducing ruminal N ammonia production and consequent urea synthesis in the liver [37]. Higher blood glucose concentration at the beginning of lactation was due to the gluconeogenesis that occurs at this stage. Moreover, the last 3 weeks before parturition and the first 3 weeks after parturition is defined as a transition period [38] characterized by depression in feed intake and incrementation in nutrient needs for gestation and lactation, which results in a negative energy balance (NEB) [39]. Higher values of blood NEFA and BHB and body fat reserve mobilization are usually indices of an adaptative response to NEB [40]. As in Monllor et al. [10], the C group had the higher body weight and higher feed intake at the beginning of lactation but had the highest BHB values compared to the AB group. However, it was not accompanied by higher values of NEFA.

## 5. Conclusions

The use of artichoke by-product silage up to 40% (on a dry-matter basis) in the diet of dairy goats over a full lactation period did not negatively affect dairy goats’ health status or milk yield, macro-composition and mineral profile. It did increase Ca concentration in milk and the Ca/P ratio, which enhance milk curd firmness. With regard to fatty acid composition, AB slightly decreased the amount of conjugated linoleic acids, increased n6/n3 ratio and worsened AI and TI indexes. AB animals did not show any sign of negative energy balance, and the AB diet decreased milk and blood urea, which may be due to the higher TP dietary amount but also to a more efficient use of protein. Regarding the significant lower economic price (−12.5% per kg DM) of artichoke by-product silage, it could be a beneficial option for local farmers. Finally, including artichoke by-product in the diet of dairy goats helps to increase the circular economy in Mediterranean areas by reducing agro-industrial wastes, improving energy and protein efficiency and producing food of high nutritional value.

## Figures and Tables

**Table 1:** Ingredients and chemical composition of the experimental diets provided to goats of the present study.

Item	Diets	
	C	AB
Ingredients (g/100 g DM)		
Alfalfa hay	37.4	5.00
Oat	16.0	14.0
Barley	8.00	7.00
Corn	9.30	8.00
Dried sugar beet pulp	7.50	7.00
Sunflower meal	3.40	3.00
Peas	3.00	3.00
Cottonseed	3.00	3.00
Soybean meal 44%	4.50	2.5
Corn DDGS	3.00	3.00
Sunflower seeds	2.00	2.00
Beans	1.40	1.10
Wheat	1.00	1.00
Soy hulls	0.50	0.40
Silage	-	40.0
Cost (EUR/kg DM)	0.32	0.28
**Chemical Composition**		
DM (g/kg FM)	882	341
	g/kg DM	
OM	920	927
EE	48.1	44.0
CP	162	152
NDF	369	444
ADF	236	293
ADL	64.1	63.4
TP	3.33	9.28
*IV*DMD	844	742
^1^ ME (Mcal/kg DM)	2.49	2.38
**VFA and Fermentative Metabolites (g/kg DM)**		
Lactate	0.00	74.7
Acetate	0.00	5.90
Ethanol	0.00	4.01
Ammonia N	0.03	0.36

C: Control diet; AB: diet that includes artichoke bract silage; DM: dry matter; FM: fresh matter; OM: organic matter; CP: crude protein; NDF: neutral detergent fibre; ADF: acid detergent fibre; ADL: acid detergent lignin; EE: ether extract; TPs: total polyphenols; *IV*DMD: in vitro dry matter digestibility; ME: metabolizable energy; VFAs: volatile fatty acids. ^1^ Metabolizable Energy.

**Table 2:** Mineral and fatty acid profile of experimental diets provided to goats of the present study.

Mineral Profile	Diets	
	C	AB
Na (g/kg DM)	1.74	4.93
Mg (g/kg DM)	2.34	2.20
K (g/kg DM)	14.05	20.7
Ca (g/kg DM)	7.30	4.67
P (g/kg DM)	3.11	3.36
S (g/kg DM)	2.88	2.30
Se (mg/kg DM)	0.134	<0.1
Zn (mg/kg DM)	43.8	73.9
Cu (mg/kg DM)	6.89	6.35
Fe (mg/kg DM)	260	156
Mn (mg/kg DM)	40.8	32.4
**Fatty acid profile (g/100 g total fatty acids)**		
C6:0	0.09	1.11
C12:0	0.15	0.06
C14:0	0.43	0.29
C16:0	16.6	15.88
C16:1 c9	0.32	0.28
C18:0	3.33	2.71
C18:1 c9	25.7	24.3
C18:1 c11	1.09	1.02
C18:2n6	44.9	41.2
C18:3n3	3.93	4.70
C20:0	0.51	0.40
C20:1n9	0.33	0.37
C22:0	0.58	0.42
C23:0	0.13	0.13
C24:0	0.36	0.41
SFA	23.0	27.0
MUFA	27.7	26.2
PUFA	49.3	46.8

C: control diet; AB: diet that includes artichoke by-product silage; DM: dry matter; SFAs: saturated fatty acids; MUFAs: monounsaturated fatty acids; PUFAs: polyunsaturated fatty acids.

**Table 3:** Comparison of body weight, milk yield and composition and somatic cell count (SCC) in goat milk, according to the effects considered from 7th to 27th lactation weeks.

Variable	n	Diet			Significance		
		C	AB	SEM	Diet	Week	Diet × Week
BW (kg)	22	44.0	42.9	0.463	n.s.	***	n.s.
Milk yield (kg/day)	22	2.13	2.21	0.067	n.s.	***	n.s.
FCM (kg/day)	22	2.42	2.45	0.084	n.s.	***	n.s.
FPCM (kg/day)	22	2.21	2.26	0.073	n.s.	***	n.s.
Fat (%)	22	4.57	4.40	0.122	n.s.	***	n.s.
UDM (%)	22	7.99	7.71	0.200	n.s.	***	n.s.
TS (%)	22	13.5	13.3	0.16	n.s.	***	n.s.
NFTS (%)	22	8.78	8.85	0.071	n.s.	***	n.s.
Protein (%)	22	3.48	3.47	0.061	n.s.	***	n.s.
True protein (%)	22	3.24	3.22	0.054	n.s.	***	n.s.
Casein (%)	22	2.82	2.84	0.050	n.s.	***	n.s.
Whey protein (%)	22	0.42	0.38	0.012	*	***	**
Lactose (%)	22	4.44	4.52	0.030	n.s.	***	*
Ash (%)	22	1.01	0.928	0.018	**	***	n.s.
Milk urea (mg/L)	22	773	687	15.3	***	***	n.s.
SCC (Log10 cell/mL)	22	2.71	2.60	0.072	n.s	***	n.s.

C: Control diet; AB: diet that includes artichoke by-product silage; SEM: standard error of mean; BW: body weight; FCM: fat-corrected milk (3.5%); FPCM: fat- and protein-corrected milk; UDM: useful dry matter content (fat + protein); TSs: total solids; NFTSs: nonfat total solids; SCC: Log10 somatic cell count. * *p* < 0.05; ** *p* < 0.01; *** *p* < 0.001.

**Table 4:** Comparison of milk mineral profile according to the effects considered from the 7th to 27th lactation weeks.

Variable	n	Diet			Significance		
		C	AB	SEM	Diet	Week	Diet × Week
Na (mg/kg)	20	378	372	3.74	n.s.	***	n.s.
Mg (mg/kg)	20	135	131	0.978	*	***	n.s.
P (mg/kg)	20	1025	1037	16.0	n.s.	***	n.s.
S (mg/kg)	20	394	376	6.3	n.s.	***	n.s.
K (mg/kg)	20	1601	1610	14.6	n.s.	***	n.s.
Ca (mg/kg)	20	1208	1242	9.6	*	***	*
Mn (µg/kg)	20	46.5	67.4	3.31	**	***	n.s.
Fe (µg/kg)	20	301	288	7.02	n.s.	**	n.s.
Cu (µg/kg)	20	72.4	85.2	2.52	**	***	n.s.
Se (µg/kg)	20	17.18	15.56	0.263	**	***	n.s.
Zn (µg/kg)	20	2624	3528	86.12	***	***	***

C: Control diet; AB: diet that includes artichoke by-product silage; SEM: standard error mean. * *p* < 0.05; ** *p* < 0.01; *** *p* < 0.001.

**Table 5:** Comparison of fatty acid composition (g/100 g total fatty acids) measured in goat milk according to the effects considered from the 7th to 27th lactation weeks.

Variable	n	Diet			Significance		
		C	AB	SEM	Diet	Week	Diet × Week
C4:0	20	1.34	1.39	0.019	n.s.	***	n.s.
C6:0	20	1.86	1.85	0.055	n.s.	n.s.	n.s.
C7:0	20	0.028	0.031	0.002	n.s.	n.s.	n.s.
C8:0	20	2.41	2.42	0.021	n.s.	***	n.s.
4-methyloctanoic acid	20	0.019	0.018	0.001	n.s.	*	n.s.
4-ethyloctanoic acid	20	0.015	0.018	0.002	n.s.	n.s.	n.s.
C9:0	20	0.042	0.041	0.002	n.s.	n.s.	n.s.
C10:0	20	8.10	8.24	0.071	n.s.	*	n.s.
C10:1 c9	20	0.027	0.029	0.003	n.s.	**	n.s.
C11:0	20	0.236	0.245	0.004	n.s.	***	n.s.
C12:0	20	3.74	3.71	0.034	n.s.	***	n.s.
9-methyldodecanoic acid	20	0.013	0.013	0.001	n.s.	n.s.	n.s.
iso C13:0	20	0.014	0.019	0.002	*	n.s.	n.s.
anteiso C13:0	20	0.040	0.041	0.002	n.s.	***	n.s.
iso C14:0	20	0.052	0.072	0.003	***	n.s.	n.s.
C14:0	20	8.49	8.45	0.046	n.s.	***	n.s.
iso C15:0	20	0.156	0.187	0.003	***	*	n.s.
anteiso C15:0	20	0.247	0.261	0.003	**	n.s.	n.s.
C14:1 c9	20	0.124	0.121	0.003	n.s.	***	**
C15:0	20	0.751	0.803	0.007	***	***	n.s.
C15:1	20	0.083	0.078	0.002	n.s.	**	n.s.
iso C16:0	20	0.202	0.230	0.003	***	***	n.s.
C16:0	20	23.9	24.5	0.101	***	***	**
C16:1 t4	20	0.022	0.032	0.004	n.s.	n.s.	n.s.
C16:1 t5	20	0.026	0.035	0.004	n.s.	***	n.s.
C16:1 t6–7	20	0.061	0.044	0.011	n.s.	n.s.	n.s.
C16:1 t9	20	0.182	0.141	0.011	*	**	n.s.
C16:1 t10	20	0.020	0.022	0.002	n.s.	*	n.s.
C16:1 t11–12	20	0.066	0.055	0.005	n.s.	*	n.s.
C16:1 c7	20	0.228	0.226	0.005	n.s.	***	n.s.
C16:1 c9	20	0.497	0.530	0.020	n.s.	***	n.s.
C16:1 c10	20	0.032	0.036	0.002	n.s.	***	*
C16:1 c11	20	0.023	0.025	0.002	n.s.	n.s.	n.s.
3.7.11.15-Tetramethyl-16:0	20	0.057	0.027	0.021	n.s.	n.s.	n.s.
Cyclo C17:0	20	0.050	0.091	0.003	***	**	**
iso C17:0	20	0.341	0.361	0.004	***	**	**
anteiso C17:0	20	0.325	0.315	0.005	n.s.	*	n.s.
C17:0	20	0.594	0.628	0.023	n.s.	n.s.	n.s.
C17:1 c6–7	20	0.045	0.046	0.002	n.s.	n.s.	n.s.
C17:1 c8	20	0.019	0.015	0.005	n.s.	n.s.	n.s.
C17:1 c9	20	0.143	0.153	0.007	n.s.	*	n.s.
Delta C17:2	20	0.021	0.028	0.009	n.s.	n.s.	n.s.
isoC18:0	20	0.042	0.042	0.003	n.s.	n.s.	n.s.
C18:0	20	14.1	14.4	0.146	n.s.	***	*
C18:1 t4	20	0.035	0.025	0.002	**	*	n.s.
C18:1 t5	20	0.034	0.027	0.002	*	**	n.s.
C18:1 t6–8	20	0.417	0.301	0.009	***	***	n.s.
C18:1 t9	20	0.408	0.304	0.008	***	***	*
C18:1 t10	20	0.572	0.452	0.017	***	***	*
C18:1 t11 (vaccenic)	20	2.13	1.37	0.059	***	***	***
C18:1 t12	20	0.550	0.449	0.011	***	***	n.s.
C18:1 t13–14	20	0.963	0.692	0.195	n.s.	*	n.s.
C18:1 c9	20	18.9	19.6	0.223	*	***	*
C18:1 c11	20	0.451	0.485	0.024	n.s.	n.s.	*
C18:1 c12	20	0.518	0.458	0.017	**	**	n.s.
C18:1 c13	20	0.120	0.095	0.007	*	n.s.	n.s.
C18:1 c14	20	0.493	0.429	0.009	***	***	n.s.
C18:1 c15	20	0.246	0.208	0.006	***	**	n.s.
C18:1 c16	20	0.023	0.021	0.002	n.s.	n.s.	n.s.
C18:2 t8.c13	20	0.138	0.121	0.006	n.s.	n.s.	n.s.
C18:2 t9.c12	20	0.027	0.030	0.003	n.s.	n.s.	n.s.
C18:2 t9.12	20	0.021	0.020	0.002	n.s.	*	n.s.
C18:2 t10.14	20	0.070	0.049	0.004	**	n.s.	n.s.
C18:2 t11.c15	20	0.048	0.044	0.004	n.s.	***	n.s.
C18:2 t11.15	20	0.012	0.011	0.003	n.s.	n.s.	n.s.
C18:2 t12.c15	20	0.030	0.029	0.003	n.s.	n.s.	n.s.
C18:2 c9.t12	20	0.321	0.288	0.004	*	n.s.	n.s.
C18:2 c9.t13	20	0.121	0.113	0.003	***	n.s.	*
C18:2 c12.15	20	0.026	0.024	0.002	n.s.	n.s.	n.s.
C18:2n6	20	2.58	2.59	0.043	n.s.	**	n.s.
CLA t9.c11	20	0.048	0.045	0.002	n.s.	*	*
CLA c9.t11 (rumenic)	20	0.843	0.602	0.017	***	***	***
CLA t10.c12	20	0.029	0.039	0.009	n.s.	n.s.	n.s.
CLA t12.14	20	0.020	0.022	0.003	n.s.	*	n.s.
C18:3n3	20	0.193	0.164	0.005	***	***	***
C18:3n6	20	0.029	0.029	0.002	n.s.	n.s.	n.s.
C20:0	20	0.241	0.232	0.009	n.s.	n.s.	n.s.
C20:1 c5	20	0.021	0.020	0.001	n.s.	*	n.s.
C20:1 c9	20	0.015	0.016	0.002	n.s.	n.s.	n.s.
C20:1 c11	20	0.052	0.043	0.003	*	*	n.s.
C20:1 c15	20	0.016	0.018	0.001	n.s.	n.s.	n.s.
C20:2n6	20	0.038	0.038	0.003	n.s.	n.s.	n.s.
C20:3n6	20	0.021	0.018	0.0022	n.s.	*	n.s.
C20:3n9	20	0.079	0.073	0.0026	n.s.	**	n.s.
C20:4n6	20	0.142	0.149	0.0067	n.s.	n.s.	*
C22:0	20	0.033	0.038	0.0022	n.s.	n.s.	*
C22:2n6	20	0.021	0.019	0.0046	n.s.	n.s.	n.s.
C23:0	20	0.023	0.403	0.0045	*	n.s.	*
C24:0	20	0.031	0.041	0.0059	n.s.	n.s.	n.s.

C: Control diet; AB: diet that includes artichoke by-product silage; SEM: standard error mean; CLA: conjugated linoleic acid. * *p* < 0.05; ** *p* < 0.01; *** *p* < 0.001.

**Table 6:** Comparison of grouped fatty acids (g/100 g total fatty acids) Indices related to cardiovascular health and desaturation activity in goat milk according to the effects considered from the 7th to 27th lactation weeks.

Variable	n	Diet			Significance		
		C	AB	SEM	Diet	Week	Diet × Week
SFA	20	67.4	68.6	0.169	***	**	*
MUFA	20	27.5	26.6	0.168	**	**	**
PUFA	20	4.93	4.64	0.064	**	***	*
UFA	20	35.9	34.6	0.12	***	**	**
OBCFA	20	3.29	3.46	0.026	***	***	n.s.
∑CLA	20	0.954	0.726	0.019	***	***	***
SFA/UFA	20	2.08	2.19	0.017	***	**	**
SCFA	20	13.8	14.03	0.115	n.s.	***	n.s.
MCFA	20	40.8	41.6	0.146	***	***	*
LCFA	20	45.3	44.3	0.183	***	***	n.s.
n3	20	0.193	0.164	0.005	***	***	***
n6	20	2.83	2.85	0.043	n.s.	**	n.s.
N6/n3	20	15.6	17.7	0.432	**	***	***
AI	20	2.02	2.09	0.018	**	***	*
TI	20	3.045	3.20	0.016	***	*	***
DI C14:0	20	0.015	0.014	0.0003	n.s.	***	**
DI C16:0	20	0.048	0.047	0.001	n.s.	*	**
DI C18:0	20	1.84	1.75	0.039	**	***	**

C: Control diet; AB: diet that includes artichoke by-product silage; SEM: standard error of mean; SFAs: saturated fatty acids; MUFAs: monounsaturated fatty acids; PUFAs: polyunsaturated fatty acids; UFAs: unsaturated fatty acids (MUFA + PUFA); OBCFA: odd- and branched-chain fatty acids; CLA: conjugated linoleic acid; SCFAs: short-chain fatty acids (C6:0 a C10:0); MCFAs: medium-chain fatty acids (C11:0 a C17:0); LCFAs: long-chain fatty acids (C18:0 a C24:0); AI: atherogenic index; TI: thrombogenic index; DI: desaturation index. * *p* < 0.05; ** *p* < 0.01; *** *p* < 0.001.

**Table 7:** Comparison of plasmatic profile according to the effects considered from 7th to 27th lactation weeks.

Variable	n	Diet			Significance		
		C	AB	SEM	Diet	Week	Diet × Week
Glucose (mg/dL)	22	50.9	51.3	0.72	n.s.	*	n.s.
Cholesterol (mg/dL)	22	103.7	99.7	1.74	n.s.	*	n.s.
Urea (mg/dL)	22	46.0	37.6	0.77	***	***	*
BHB (mmol/L)	22	0.50	0.42	0.021	*	***	n.s.
NEFA (mmol/L)	22	0.56	0.60	0.031	n.s.	***	n.s.
Haematocrit (%)	22	30.7	30.6	0.32	n.s.	***	n.s

C: Control diet; AB: diet that includes artichoke by-product silage; SEM: standard error of mean; BHB: β-Hydroxybutyrate; NEFAs: nonesterified fatty acids. * *p* < 0.05; *** *p* < 0.001.

## Data Availability

The data presented in this study are available on request from the corresponding author.
